# Multimodal Network Architecture for Shared Situational Awareness amongst Vessels

**DOI:** 10.3390/s21196556

**Published:** 2021-09-30

**Authors:** Amina Seferagić, Jetmir Haxhibeqiri, Paolo Pilozzi, Jeroen Hoebeke

**Affiliations:** 1 IDLab, Ghent University-Imec, Zwijnaarde Technologiepaark 126, B-9052 Ghent, Belgium; Jetmir.Haxhibeqiri@UGent.be (J.H.); Jeroen.Hoebeke@ugent.be (J.H.); 2 Intelligent Mobile Platforms (IMP) Research Group, Department of Mechanical Engineering, KU Leuven, Andreas Vesaliusstraat 13, 3000 Leuven, Belgium; Paolo.Pilozzi@KULeuven.be

**Keywords:** Wi-Fi, IEEE 802.11, maritime communications, application-network integration, shared situational awareness, multimodal communications

## Abstract

To shift the paradigm towards Industry 4.0, maritime domain aims to utilize shared situational awareness (SSA) amongst vessels. SSA entails sharing various heterogeneous information, depending on the context and use case at hand, and no single wireless technology is equally suitable for all uses. Moreover, different vessels are equipped with different hardware and have different communication capabilities, as well as communication needs. To enable SSA regardless of the vessel’s communication capabilities and context, we propose a multimodal network architecture that utilizes all of the network interfaces on a vessel, including multiple IEEE 802.11 interfaces, and automatically bootstraps the communication transparently to the applications, making the entire communication system environment-aware, service-driven, and technology-agnostic. This paper presents the design, implementation, and evaluation of the proposed network architecture which introduces virtually no additional delays as compared to the Linux communication stack, automates communication bootstrapping, and uses a novel application-network integration concept that enables application-aware networks, as well as network-aware applications. The evaluation was conducted for several IEEE 802.11 flavors. Although inspired by SSA for vessels, the proposed architecture incorporates several concepts applicable in other domains. It is modular enough to support existing, as well as emerging communication technologies.

## 1. Introduction

Transport over water is an important part of freight transport, where around 80% of the world’s trade is shipped over sea [1]. As is the case in almost any transport sector, transport over water is undergoing an evolution towards unmanned vessels, supported by shore/remote control centers, and in the next stage autonomous vessels. To support this evolution, work is being done to connect the actors in this quite specific environment in light of the Industry 4.0 evolution by applying Internet of Things (IoT) technologies.

The main purpose for connectivity is of course enabling actors to have access to the necessary information to develop situational awareness, and more importantly, a shared version thereof, so that data critical to recognizing situations can be shared from and to the actors of the environment, even in real-time. Therefore, the underlying network architecture for supporting such communication is an active field of research, both in the maritime and inland waterway setting.

It should be clear that communication between vessels offshore has been more challenging as compared to application fields on land, simply because one cannot rely on the vast and dense communication infrastructure available on land. For example, ships in need of exchanging information can be far at sea, hundreds of miles apart. Ships in the vicinity of each other performing collaborative tasks on international waters will need to share resources (like radar, lidar, sonar, or other means of perceiving the world around them) and be connected to information systems (like for weather, traffic, currents, etc.). Furthermore, in a transition to fully autonomous vessels, and even long after, ships need to be connected to control centers (mostly on land), monitoring vessel actions as well as having the capability to take control when a situation calls for it. Therefore, these centers will need the tools to be aware of the situation a vessel is in while operating vessels as if they are locally at the helm of those vessels.

Advances in connected vessels for Shared Situational Awareness (SSA) are today constrained by a limited set of communication methodologies. One example of a broadly used system for SSA is Automatic Identification System (AIS). However, AIS has very limited capabilities regarding the sharing of vessel states and their intentions. Being an older technology, security is lacking (for example spoofing, i.e., pretending to be another vessel), update rates are lacking (not faster than once per 2 s and in worst case once per 12 min), there are scaling problems (for example relevant to busy harbors), and correctness guarantees and trust is missing (only 30% of AIS messages are found to be correctly representing a vessel’s intentions and states [2,3]). Moreover, the strong connection between the AIS protocol and the physical (wireless) link used to transmit and receive messages makes it a less than optimal solution to support future developments in communication for unmanned (autonomous) shipping, requiring SSA. That is, additional connectivity solutions are required to complement AIS.

Independent of the information content shared for SSA, the channels over which that information can be shared require special attention. Next to Very-High Frequency (VHF) radio broadcasting (for AIS), in practice, vessels make use of cellular networks, satellite networks, and point-to-point networks (e.g., Wi-Fi mesh) to interconnect, and this to share information, diagnose problems, remotely control assets, and collect big data. However, even with these connectivity solutions, vessel operators face significant limitations related to data rates, coverage, topologies, latency, etc. Hence, to cover for the varying communication needs across edge, fog, and cloud solutions, there are many challenges to consider. For instance, satellite communication (although omnipresent) has low bandwidth, high latencies, and a high usage cost. Cellular networks, only available in near-land and inland waters, can have poor coverage in low-populated regions and can demonstrate frequent handover failures. Wi-Fi mesh (although high in bandwidth) is a localized technology, operating in unlicensed spectra and thus prone to unwanted/unexpected interference.

Obviously, any communication technology has drawbacks and advantages and finds appropriate use depending on circumstances. Fall-back and collaborative mechanisms to ensure robust, reliable, and secure communication are therefore a necessity, employing the right technologies to the situation at hand, preferably collaboratively and in a transparent way: The SSA application in need of connectivity should not be responsible for establishing the connectivity, optimizing and maintaining it, but only using it. To this end, the following main contributions are made in this paper. (i) We design and implement a multimodal communication framework to support SSA for vessels that is (a) self-organizing, whilst being transparent to the higher layer applications, (b) that provides relevant feedback on the communication capabilities to the applications and (c) that is sufficiently modular to incorporate new technologies when they appear on the market (e.g., the upcoming long-range Wi-Fi standard IEEE 802.11ah). (ii) We design and implement a centralized discovery framework that enables automatic communication bootstrapping between vessels. (iii) We design and implement a simple time-aware scheduling mechanism that makes use of application-network integration framework [4] to fulfill the applications’ latency requirements. Finally, (iv) we evaluate the design in three representative use cases.

More specifically, we introduce an abstraction layer in between layers 2 and 3 of the Open Systems Interconnection (OSI) communication stack that dynamically bootstraps communication using any and all of the available physical network interfaces. This layer informs applications regarding available networking capabilities (in real-time) enabling the application to adapt to the network (e.g., compress data or filter some out when links degrade or start new data streams when better links become available, etc.) while providing services to the application based on its needs as a best effort (e.g., regarding prioritization, bandwidth shaping, etc.). As such, our concepts enable multimodal communication, flexibly and transparently, providing traffic management without connectivity interrupts for the application layer. Our approach is both technology and vendor/manufacturer agnostic and can cope with various communication channels with fluctuating quality that might only be temporarily available. By better understanding network behavior, our approach enables applications to optimize the data transfer, as well as to have more control over the multimodal network’s Quality of Service (QoS).

In this paper, we describe the design and proof-of-concept implementation of the network architecture for SSA and evaluate this solution in three scenarios, considered representative for SSA use cases for vessels in the maritime, estuary, and inland waterway context. These scenarios are: (i) automatically bootstrapping the communication between moving vessels depending on their location, (ii) automatically adapting the network behavior in real-time based on the application requirements, and (iii) automatically adapting the application behavior in real-time based on the current network conditions.

In the next section (Section 2), we discuss related work, i.e., the state-of-the-art. Section 3 describes challenges and requirements related to SSA for vessels. Section 4 presents the design and implementation of the communication architecture, which is evaluated in Section 5 for the three aforementioned scenarios. Finally, conclusions are given in Section 6.

## 2. Related Work

Despite the advent of radio communication being largely motivated by maritime applications, advancements in maritime communications are significantly lagging behind their terrestrial counterparts (e.g., IEEE 802.11, 3GPP cellular). While wireless communications have undergone significant advancement and breakthroughs, only in recent years maritime communication has slowly been gaining momentum in modernizing mobile communication services. As such, the International Telecommunication Union (ITU) introduced AIS to provide vessel identification, location reporting, and tracking in order to enable the exchange of navigational data among ships and between ships and shore stations with the aim to improve situational awareness over voice, sight, and radar [5]. However, AIS has limited data communication capacity and lacks a flexible architectural framework for addressing current and upcoming aspects of maritime IoT applications and services, and can be deemed as a primitive maritime communication system [6].

Although there is some interesting literature on maritime communication technologies utilizing particular terrestrial and satellite communication technologies as reviewed in [6,7], there is very limited research available on multimodal maritime communication systems. Three recent research papers propose conceptual network architectures for maritime use-cases analyzing the overall operation of the system, but do not provide concrete implementations or evaluations of such systems [6,8,9]. They all present a maritime Machine-Type Communication (MTC) concept dedicated to the maritime IoT and outline the maritime MTC requirements on the ubiquity, heterogeneity, interoperability, service-centricity, traffic nonuniformity, and scalability, to name a few. They address these requirements through an MTC system design, based on the international VHF maritime mobile spectrum recently allocated for maritime MTC by ITU to enable maritime IoT. Wang et al. [8] point out challenges, advice, and pitfalls to avoid in future development and standardization of the maritime communications, but do not present an implementation or evaluation of such system. In addition to the unified network architecture as presented in [8], Zhang et al. [6] propose the spectrum sharing and interference management mechanisms for VHF maritime mobile spectrum. Xia et al. [9] also propose an analogous unified network architecture, further defining its three functional entities: Network Controller, Maritime Application Server, and Control Station. Additionally, they propose an air interface design that includes four types of air interfaces, employing single-carrier waveforms for power efficiency and simplicity, and sharing the same transmission time structure that is organized into frames.

Mu et al. [10] proposed an integrated wireless communication architecture design that tries to provide maritime customers ubiquitous services by integrating heterogeneous underlying wireless networks. Solutions for addressing key issues such as quality, security, and mobility are covered in this architecture with a more detailed discussion of seamless handover. The proposed design is not implemented or validated in this study.

Maritime communication systems are briefly but concisely reviewed by Robinson et al. [11]. Satellite communications including Low Earth orbit (LEO), Medium Earth orbit (MEO) and Geostationary orbit (GEO), point-to-point wireless links, as well as research communications (i.e., evaporation ducts and troposcatter), are compared in this study in terms of range, bandwidth, latency, and cost. A more detailed, comprehensive review of hybrid satellite-terrestrial maritime communication networks, including communication requirements, state-of-the-art networks, and enabling technologies is published by Wei et al. [7]. They categorized the enabling technologies into three types, namely enhancing transmission efficiency, extending network coverage, and provisioning maritime-specific services. They illustrated and compared the technologies in terms of their objectives, methods, and characteristics of used maritime communication networks.

Several studies have evaluated existing wireless technologies in the maritime domain. Allal et al. [12] conducted a case-study in the Strait of Gibraltar in order to study the reliability, cost-effectiveness and availability of satellite communication, radio VHF, radio Ultra High Frequency (UHF), radio High Frequency (HF), radio Medium Frequency (MF), AIS and Long Range Identification and Tracking (LRIT). They quantified and classified the data to be transferred, the identification of ship mobility environment challenges, and proposed mobile WiMAX (IEEE802.16e-2005) as a reliable, secure, and cost-effective maritime communication carrier in the strait of Gibraltar where conventional and autonomous ships will co-exist. Choi et al. [13] also evaluated WiMAX (IEEE 802.16j) in terms of Bit Error Rate (BER) under various sea states in vessel to vessel communication. Ref. [14] compares different routing protocols in a delay tolerant WiMAX mesh network in maritime environment.

Hassan et al. [15] propose a heterogeneous communication framework using 6G, LEO and an Unmanned Aerial Vehicle (UAV) to provide global connectivity to maritime users. They proposed UAV as an aerial backhauling and a relay medium between marine users and LEO satellite constellation. Other studies [16,17] present Long Term Evolution (LTE)-Maritime, a research project in the Republic of Korea to develop the communication infrastructure for providing higher data rates with coverage around 100 km from shoreline based on LTE technology.

Finally, Aliaj et al. [18] developed a software platform for performing wireless maritime networking experiments. The platform brings together two features. First, a novel middleware application, called Dedalus, is used for monitoring and controlling experiments, and secondly, several implementations of popular protocols that perform ad hoc routing, delay-tolerant routing, information-centric networking, and encryption.

This paper takes into account maritime communication requirements as detailed in several previous conceptual studies [6,8,9] and presents the design, implementation, and validation of a multimodal maritime communication framework for enhancing shared situational awareness for vessels. No research to date has yielded a concrete system that is environment-aware, service-driven, and technology-agnostic.

## 3. Requirements and Challenges

To achieve an enhanced situational awareness of vessels, it is necessary to provide connectivity to various types of maritime applications and services. This section first summarizes the general requirements and challenges that such a maritime communication system faces. Then, it presents more focused technical requirements and the scope of this paper in terms of addressing the requirements. Finally, it summarizes the challenges in implementing a multimodal and flexible communication system for the maritime domain.

### 3.1. Maritime Communication Requirements

Recent studies have comprehensively categorized the maritime communication requirements, as detailed below. Zhang et al. [6] distinguished three key characteristics a maritime communication system requires, namely (i) ubiquitous Connectivity and Service Continuity, (ii) traffic nonuniformity, and (iii) radio spectrum internationality. In addition to these three, Wang et al. [8] and Xia et al. [9] distinguish 5 more requirements as follows: (iv) service-centricity and adaptability, (v) device heterogeneity, (vi) simplicity and reliability, (vii) capacity and scalability and (viii) interoperability.

#### 3.1.1. Ubiquitous Connectivity and Service Continuity

To ensure continuous and unbroken access to maritime services among vessels and vessel-to-shore on a global scale in open oceans, including remote Polar regions, a unified cooperative service network that can provide seamless roaming with undisrupted services across organizational, regional, and national boundaries is needed.

#### 3.1.2. Traffic Nonuniformity

The highest density of vessels is present in and around ports, near-shore and waterways, whereas deep-sea vessels are sparse in density. Hence, an effective and adaptable communication solution is needed to handle this heavy traffic load disparity.

#### 3.1.3. Radio Spectrum Internationality

Maritime communication has traditionally used a dedicated radio spectrum for communications, which is regulated by national standardization bodies, calling for a dedicated international frequency band.

#### 3.1.4. Service-Centricity and Adaptability

Having in mind that maritime applications and services vary from simple location reporting to route exchange and remote control (e.g., for autonomous vessels), maritime communication network needs to cooperate with other networks (e.g., Internet, owner’s intranet, etc.) and offer amorphous services that can adapt to a wide variety of maritime applications and support the changing demands. Hence, both network configuration and communication resources must be flexible and adaptive to varying services. In addition, the network needs to provide security and ensure that only authenticated vessels can access certain services, and vice versa.

#### 3.1.5. Device Heterogeneity

Maritime communications include a wide variety of end devices ranging from low-cost low-power sensors to high-end devices for large vessels. Ergo, the communication network needs to support different communication and processing capabilities these devices can provide.

#### 3.1.6. Simplicity and Reliability

Given that maritime equipment needs to endure harsh environmental conditions, reliability and robustness are of paramount importance. To reap the full benefits of maritime communications, communication devices will have to be mandated to all vessels. Hence, the system has to be designed with low cost in mind and needs to be simple as simpler systems are easier to manufacture and maintain.

#### 3.1.7. Capacity and Scalability

Maritime communication systems need to support the ever-growing maritime traffic, despite the scarce radio spectrum. In addition to spectrally efficient communication technologies, maritime communication systems need to be flexible in more than one way, enabling future-proof scalable deployments.

#### 3.1.8. Interoperability

The ability of different maritime applications and services to exchange data efficiently and cooperatively needs to be ensured, having in mind that maritime applications are provided by different companies and organizations in the maritime industry. Given that maritime applications and services need to be available to all involved hosts, regardless of manufacturer, owner, or origin, the underlying communication system needs to enable interoperability.

### 3.2. Implementation Challenges

Given the wide variety of both services needed and communication technologies in use, one of the major challenges in realizing a communication solution for comprehensive situational awareness in the maritime domain is communication bootstrapping. Vessels in remote regions do not need to exchange much data, but do need to send status reports every once in a while, and can only use satellite communications for such long-distance communication. In case of multiple vessels in the vicinity to each other in offshore areas, in absence of network infrastructure an ad hoc network needs to be established in order to exchange more data between them. *Vicinity* in open seas may mean anything from a hundred meters to more than a kilometer. When closer to the terrestrial communication infrastructure nearshore, vessels may use cellular communication. Having in mind ubiquitous connectivity and service continuity requirements, communication bootstrapping should be smooth, without requiring the application to explicitly select a specific technology, which would disrupt ongoing communications and services. The use of multiple communication technologies (also referred to as multimodal communication), including the presence of multiple IEEE 802.11 flavors, comes with challenges in terms of routing and traffic handling without any connectivity interrupt for the application layer.

To dynamically bootstrap multimodal communications, vessels need to have a technology-agnostic discovery mechanism and be able to detect other vessels and their communication capabilities and services they offer. They need to (be able to) establish an ad hoc communication network (like IEEE 802.11 ad hoc mode) dynamically when possible and needed, and make use of this network in addition to, or instead of, other networking options (e.g., cellular, satellite, IEEE 802.11 managed mode). We distinguish two options regarding ad hoc communication bootstrapping, as follows:

- *Application is already communicating* over another link (e.g., cellular) with a lower data rate. Establishing a direct ad hoc IEEE 802.11 link may enable a higher data rate, in which case the application may send the same sensor stream at a higher data rate over the new link, or add a new stream, while still prioritizing between the streams.
- *Application is not communicating*. The presence of a new link with certain properties may trigger the discovery of additional services and start the data exchange in one or both directions.

Wi-Fi mesh networking is not a novel concept and is already in use within some organizations in the maritime domain. However, establishing a mesh network between vessels owned by different organizations is not seamless. Vessels need to discover each other, discover the services each of them offers and connect to each other while providing service continuity in case they already communicated over another link. In addition, same channel meshing may introduce both intra- and inter-network interference, as well as limit the network capacity, especially in ports and waterways where many vessels coexist in the same geographical area. To make the most out of Wi-Fi mesh, distributed and adaptive network configuration is needed.

Requirements listed in Section 3.1 mandate bootstrapping the connectivity between assets at different levels (edge-fog-cloud) and from different manufacturers and owners, over various communication channels that can have fluctuating quality and that might only be available temporarily. To set up a distributed, shared environment model over such a dynamic communication environment, we need to move towards flexible and secure multipath communication solutions. At the same time, deeper insights in the communication behavior are needed to optimize data distribution and adjust data reductions at the application level. To be able to optimally utilize any or all of the communication links available at a certain time, applications in vessels need to be aware of current network capabilities and link properties for every existing link. Hence, the concept of application-network integration should be adopted. An application should have the means to find out what are the properties of the available links in real-time, and the network should have the means to find out what needs do applications have in terms of bandwidth and time, in order to provide appropriate QoS to them, if possible.

Low-cost IP-like technologies would enable access to services in the cloud over the Internet, making the communications service-driven and technology-agnostic, irrespective of the equipment available and vessel ownership. Costly proprietary solutions, however effective, may not be suitable and affordable to small organizations or individuals which disables the global shared situational awareness.

In summary, although some building blocks are available in the state of the art, there are significant gaps in:

- Tackling multimodality, and thus multipath communication, in a dynamic environment that also involves ad hoc networking,
- Closely monitoring the communication capabilities using telemetry to follow-up in real-time and with limited overhead the behavior of the communication network,
- Closing the loop between the communications and the applications, with communication delivering a more specific treatment of data based on requirements provided by the application and the application receiving feedback on the communication capabilities to enable adjustments.

## 4. Communication Architecture

We target a modular architecture considering the following aspects:

- Communication stack,
- Communication bootstrapping, and
- Application-network integration.

Communication stack handles multimodality aspect of communications, as well as QoS through time-aware scheduling. Bootstrapping handles the discovery of neighboring vessels and triggering direct data exchange. Application-network integration takes care of providing application requirements to the network, as well as providing feedback from the network to the applications.

### 4.1. Communication Stack

**Figure 1:**
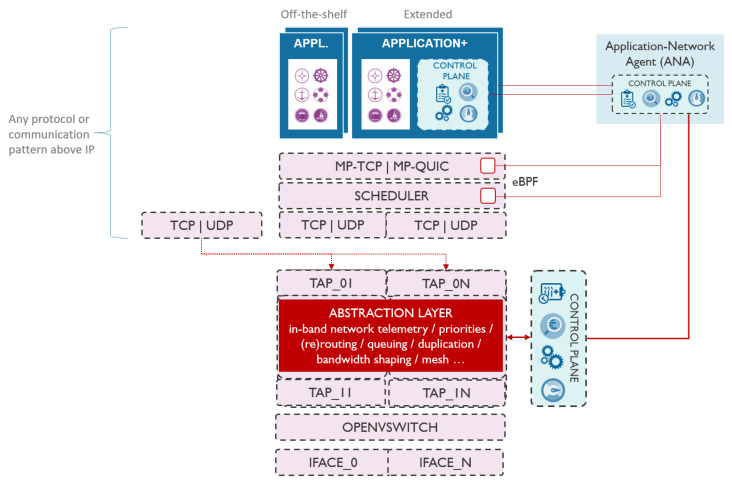
Communication architecture with the abstraction layer for traffic handling.

To enable seamless multimodal communication via various network interfaces, we propose introducing an abstraction layer in userspace that is transparent to the higher network layers and handles multimodal communications entirely. As illustrated in Figure 1, the abstraction layer is located between layer 2 and layer 3 of the network stack. All traffic generated by applications is passed via the abstraction layer, which enables it to take care of traffic handling, including in-band network telemetry. This makes the abstraction layer a de facto processing element between the application and the physical interfaces. In presence of multiple network interfaces (e.g., cellular, IEEE 802.11 and long-range), abstraction layer logic can reroute the traffic over another interface, duplicate the traffic to another interface, prioritize between different traffic streams, shape the bandwidth according to the application requirements, etc. Such architecture enables smooth communication technology switch without disrupting the ongoing communications and connections to services, even in presence of unstable or temporary links.

In this context, the abstraction layer has a threefold role, bootstrapping communication using the information from the neighbor discovery mechanism, handling in-band network telemetry, and improving QoS. Bootstrapping the communication includes detecting the vessels in the neighborhood as detailed in Section 4.3, and using this information to trigger (secure) information sharing in the context of situational awareness. In-band network telemetry is used to measure the performance of the network in real-time, per-packet basis, hop-by-hop, and end-to-end without introducing much overhead to the network [19]. Finally, QoS is currently handled through an adaptive time-aware scheduling mechanism that ensures meeting the latency requirements of the applications, as described in Section 4.2. QoS can be further improved by introducing other state-of-the-art mechanisms.

To evaluate the proposed architecture, we opted to implement the abstraction layer in user-level using Click modular router framework [20]. To avoid processing the traffic twice, in kernel and in user-level, we used Open vSwitch (OVS) to bypass the part of the kernel stack and divert the traffic via the abstraction layer only. As shown in Figure 1, the abstraction layer creates 2*N* TAP interfaces in Kernel, *N* in northbound direction (i.e., towards the application) and *N* in southbound direction (i.e., towards the physical devices), where *N* is the number of used physical network interfaces. OVS bridges each southbound TAP interface with its corresponding physical interface. On the other hand, the two corresponding southbound and northbound TAP interfaces are bridged between each other using the abstraction layer itself. When OVS bridges an incoming layer 2 packet from the physical network interface to the southbound TAP interface, the kernel forwards that packet to the abstraction layer in userspace. The packet is processed accordingly by the abstraction layer and it is passed further to the respective northbound TAP interface, and further to the application. In this case, no IP addresses are assigned to the southbound TAP interfaces, nor to the physical network interfaces. IP addresses are configured for northbound TAP interfaces only, and can either be set manually or gotten from a DHCP server in the network. There will be no IP addresses collisions as long as each interface is connected to different network and uses different subnetwork address, which is the case in vessel communication. In case of vessel communication, each interface will be connected to different network, e.g., one interface might be connected to celullar network while the other to the Wi-Fi based ad-hoc network. In the other direction, traffic coming from the applications will end up in the abstraction layer via the northbound TAP interface(s). The abstraction layer can then process the traffic and send it out through the corresponding southbound TAP interface(s) that are bridged to physical interfaces. This implementation enables the abstraction layer to control the traffic in user-level while avoiding additional delays as there is no interfacing with the kernel stack.

### 4.2. Time-Aware Scheduling

IEEE 802.11 and LTE standards define several traffic classes to prioritize traffic based on application requirements. IEEE 802.11 uses variants of random access scheduling, where each traffic class is assigned a queue and Carrier-Sense Multiple Access with Collision Avoidance (CSMA/CA) is used on each queue on a node. For example, some IEEE 802.11 variants (e.g., IEEE 802.11e, IEEE 802.11ah) use Enhanced Distributed Channel Access (EDCA) that defines four access classes: voice (VO), video (VI), background (BK) and best effort (BE). The higher the priority, the more chances to access the wireless channel [21]. However, operating at a high priority class does not always minimize delay, especially when Wi-Fi and LTE coexist in unlicensed bands. The class selection of one system impacts the performance of the other, and selecting a lower priority class may result in a lower delay under certain contention and class selection conditions [22].

The concept of Time-Aware Shaper (TAS) was recently standardized by the IEEE 802.1 Time Sensitive Networking (TSN) working group in order to provide deterministic latency guarantees. However, TAS requires precise time synchronization in all network switches, which is why it has not been standardized for the wireless domain yet. To this end, research effort has been shifting towards bringing TSN into wireless networking and providing low-overhead, high precision time synchronization in IEEE 802.11 networks [23,24].

For these reasons, instead of using vague priority classes, we designed time-aware scheduling as a feature in the abstraction layer that dynamically differentiates streams, and implemented a proof-of-concept solution using Click modular router framework [20]. Based on application requirements, the time-aware scheduler first categorizes packets by period to a predefined number of queues $k_{max}>1$, and then time-schedules pulling from the queues proportionally to the queues’ loads and considering the strictest application requirements of flows in each queue. Ergo, queues can only emit packets in their assigned time intervals which are determined based on the queue load and the application requirements. This enables deterministic scheduling of time-critical frame transmissions while avoiding contention with low priority frames so that background traffic does not impact the time-critical traffic, and vice versa.

**Figure 2:**
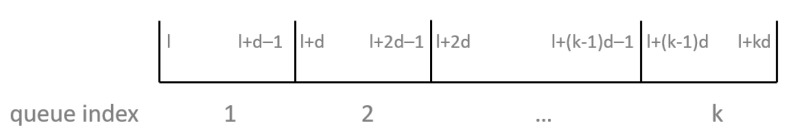
Time-aware queues.

For each packet, the time-aware scheduler first extracts the required latency from the application requirements based on flow information (IP and port). As illustrated in Figure 2, it classifies packets according to their required latency into queues, where each queue represents a latency interval [l+(k−1)d,l+kd−1] (except the last one indexed $k_{max}$ which represents [l+(k−1)d,l+kd]), where *l* is the lowest latency requirement encountered at runtime, k∈N index of the queue in range $\left[1,k_{max}\right]$, and $d=\left(L-l\right)/\left(k_{max}-1\right)$ the time interval used to linearly split the time between lowest required latency *l* and largest required latency *L*. The scheduler updates the latency values *l* and *L* with each packet, calculates *d*, and pushes the packet to the appropriate queue based on its latency requirement, which concludes the categorization.

The time-aware scheduler pulls from each queue for the time proportional to the queue load, while respecting the latency requirements. In other words, a queue does not wait to be pulled from for a time longer than the lowest latency requirement of all flows served by that queue. Time to assign to each queue is determined as follows. Time-aware scheduler determines the load of each queue in bytes per second using the number of packets in the queue and flow information from the application requirements. The queue that serves non-monitored traffic (i.e., the traffic that has no application requirements defined) is considered the least priority queue, and the load allocated for that queue is set to half of the value of the first non-empty larger priority queue. Once the traffic load of each queue is known, time-aware scheduler calculates time $T_{k}=L_{k}x$ to assign to each queue *k* proportionally to its load $L_{k}$, where *x* is proportion coefficient calculated from:

(1) $$\sum_{k=2}^{k_{max}}L_{k}x\leq l,$$

and *l* is the smallest required latency encountered in runtime. This ensures that even the queue k=1 which contains the flow with the strictest latency requirement *l* will never be blocked by other queues for longer than *l*. As a consequence, all other queues that contain flows with less strict latency requirements will be served in a timely fashion. This results in serving each queue as frequently as the latency requirements of the packets in that queue need while distributing the total channel time fairly between all queues (i.e., proportionally to the queue loads). The overall period of iterating through all queues depends on the latency requirements of the packets but is not necessarily less than *l*. However, the sum of all times assigned to queues 2 through $k_{max}$ will be less than *l*, considering that *l* is the lowest latency requirement and queue indexed 1 will serve packets with such requirement, so queue 1 will never wait for more than *l* to be pulled from.

### 4.3. Communication Bootstrapping

**Figure 3:**
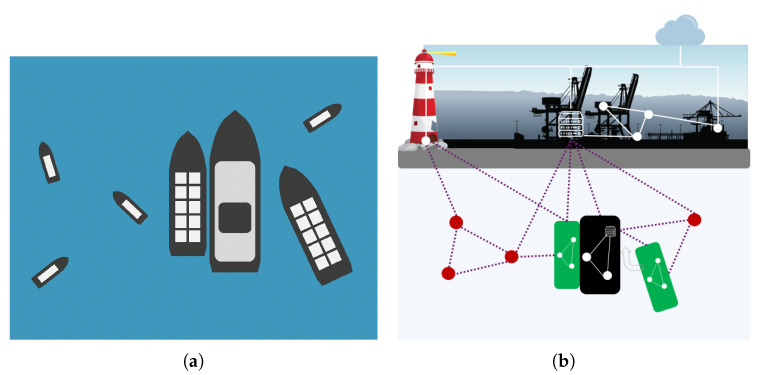
Bootstrapping the communication among neighboring vessels: (**a**) illustration of a docking scenario and (**b**) topology illustration of mesh network among neighboring vessels.

Vessels in the vicinity to each other may wish to exchange more information than standard location/intentions messages and would benefit from establishing a direct line of communication. A perfect example of this scenario is a vessel docking to another moving vessel. In order to dock successfully, the docking vessel needs to get very frequent updates (measuring in tens or hundreds of milliseconds) on the position and movements of the other vessel, currents, and other situational data, and cannot tolerate the delay by communicating over the Internet. Figure 3 illustrates this scenario.

In order to bootstrap the communication, vessels first need to discover each other, and use this information to trigger (secure) data exchange, possibly via direct mesh links. We identified three discovery strategies: fully centralized, hybrid, and decentralized. All three are discussed below.

#### 4.3.1. Centralized Discovery

**Figure 4:**
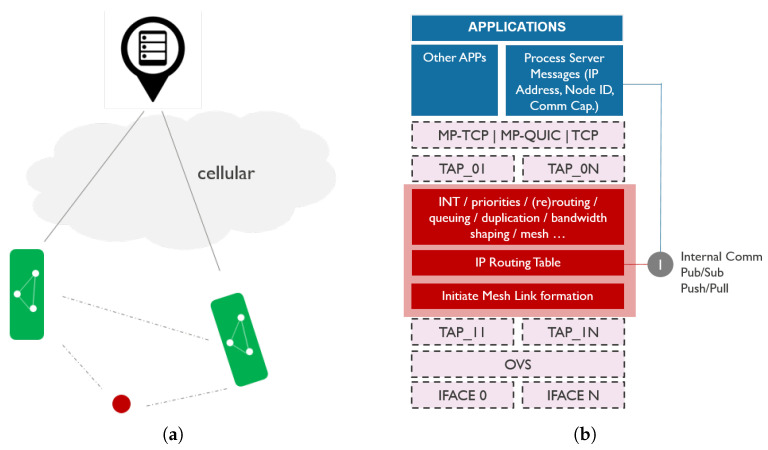
Centralized discovery including (**a**) schematic representation of network topology and (**b**) communication stack design.

Centralized discovery, illustrated in Figure 4, relies on a known central server that collects information on all vessels. Vessels publish their own information to the central server over an existing link, including their identification, location information, and communication context (e.g., mesh capabilities). Optionally, they can also share information on data sources or services they offer. Vessels that wish to set up a direct link with other vessels in the neighborhood can then pull the information on neighbors from the central server, and learn about their location and communication capabilities in order to trigger the link setup.

We modeled the communication capabilities message as a structure in JavaScript Object Notation (JSON) format shown in Listing 1. The structure consists of two main parts, *general information* and *interface information*. *General information* contains a unique identification of the vessel (i.e., Maritime Mobile Service Identity (MMSI)), its global position, and the number of communication interfaces at its disposal. *Interface information* contains a substructure for each interface. This substructure includes the type of the interface and its properties. Properties include IP configuration, range of the used wireless technology, interface name and its Media Access Control (MAC) address. For mesh point interfaces, properties also include mesh ID (i.e., Service Set Identifier (SSID)) and channel information, which is enough for the neighboring vessel to connect. In addition to this, properties of mesh point interfaces also include an active flag to indicate if the interface is currently in active use, and a role that can take values transmit (TX), receive (RX) or ANY. TX denotes interfaces that are only used to connect to other vessels in order to transmit data, RX interfaces that continuously listen for incoming connections, and ANY interfaces that are used bidirectionally.

**Listing 1:**
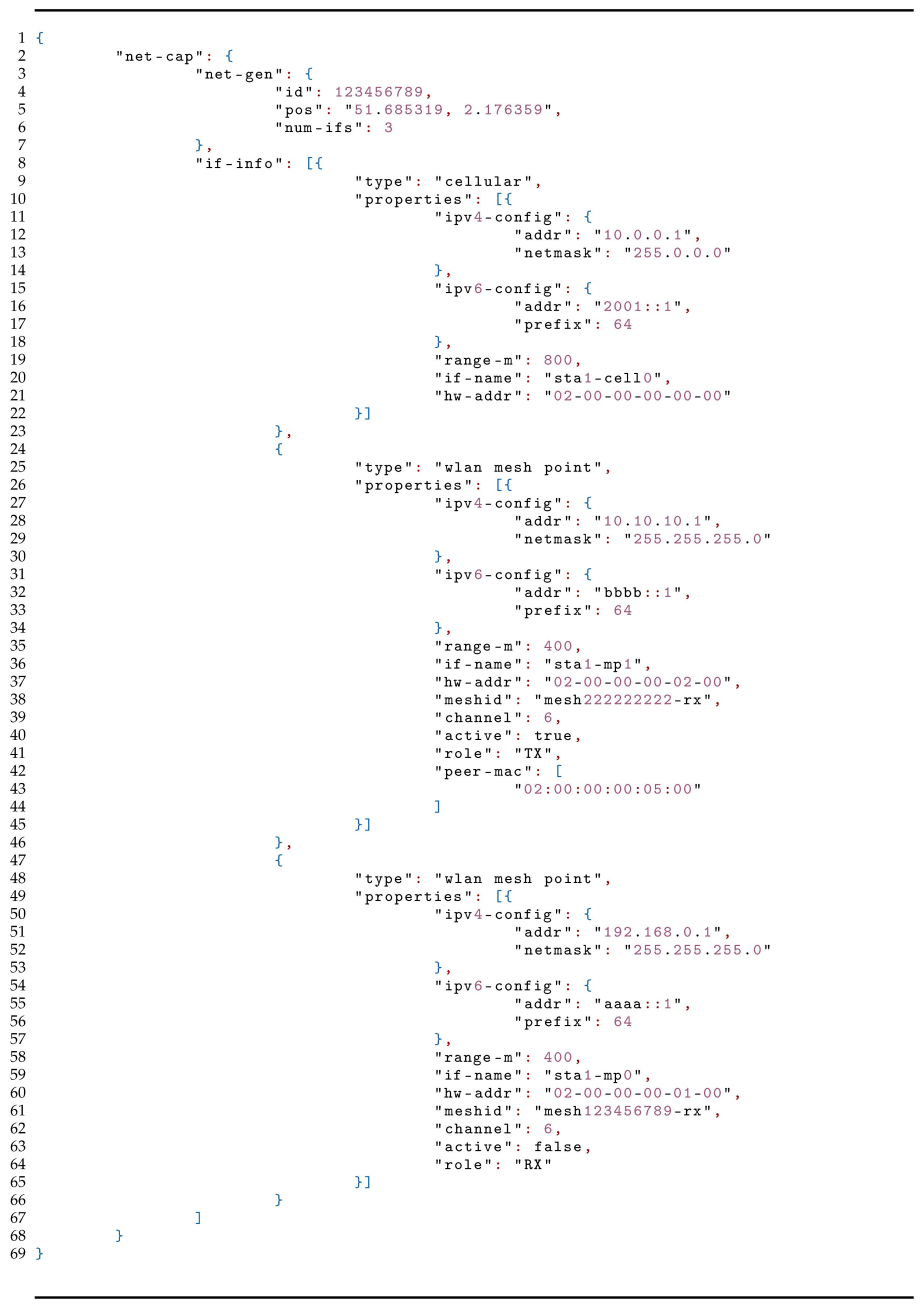
Communication capabilities message for publishing to the central server: example.

A vessel can use any centralized framework (e.g., publish/subscribe, request/reply) to exchange the communication capabilities messages via a central server over the Internet. Every vessel periodically publishes its communication capabilities, and other vessels can subscribe to receive updates. When vessel A receives the JSON structure of a neighboring vessel B, it compares the location of B to its own location, and based on the range information can conclude if it is in the communication range of the vessel B or not. If it is in the range, vessel A can join the Wi-Fi mesh network of the RX interface of vessel B. This link establishment is handled by the abstraction layer. In order to keep the network scalable, we opted to use two radios for mesh, one for TX and one for RX. If the same radio was used for both incoming and outgoing traffic, the throughput would be halved as the radio cannot transmit and receive at the same time, and would have to switch roles from TX to RX and vice versa frequently in runtime. In addition, every mesh link would have to use the same radio channel. This means when one radio is transmitting, all its neighbors must be in listening mode. This problem is amplified across the mesh, and after a few hops, the architecture could be slowed to the point where it no longer efficiently supports communication. Using two radios alleviates the aforementioned problems. RX interface will never switch channels unless it wants to avoid other congested channels in the neighborhood, but the TX interface will switch channels each time it wants to transmit data to another neighbor that listens on another channel.

The downside of this approach is the dependency on the central server in the architecture. If the vessel has no connection to the server, it cannot perform the neighbor discovery, hence cannot bootstrap the communication. Unless multipath transport protocols are used, the information on the existence and properties of the newly established link need to be propagated to the application as well, so that it can start using it (i.e., send traffic to the appropriate IP address). The application can either subscribe to the communication capabilities itself and learn about the new link from there, or it needs to interface with the abstraction layer to get the link information from there. On the other hand, the abstraction layer may reroute or duplicate the existing traffic over the new link and feed the link properties back to the application via in-band network telemetry. This may trigger the application to start using more services or switch to the new link entirely. In addition, if multipath transport protocols are used, establishing a new link will result in more bandwidth available to the applications.

#### 4.3.2. Hybrid Discovery

**Figure 5:**
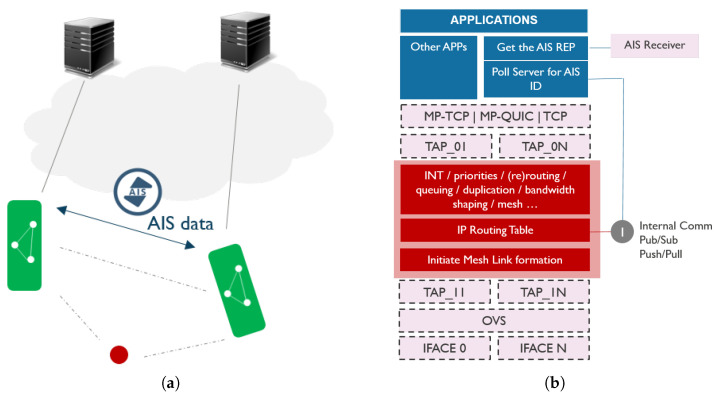
Hybrid discovery including (**a**) schematic representation of network topology and (**b**) communication stack design.

Hybrid discovery is schematically represented in Figure 5. It relies on the existing infrastructure in the maritime domain. Vessels owned by a certain organization usually publish their communication context, status, and services to the central server of that organization. Moreover, vessels typically use AIS to share basic information among each other and with the central entities on the shore. However, AIS data can also be used to trigger bootstrapping the communication between neighboring vessels. Every vessel periodically broadcasts its navigational information (i.e., longitude, latitude, heading, time, speed, status: anchored or under power) via AIS. When vessel A receives such AIS message from vessel B, vessel A may query the server to fetch additional information on communication capabilities or services of vessel B based on its identifier learned from the AIS message and establish a direct link (e.g., mesh) to each other.

If vessels do not have access to the same central servers, they may not be able to retrieve the information necessary to establish the new link. This may well be the case for the vessels owned by different organizations. In this case, vessels may exchange the information necessary for the new link setup via extended AIS messages. However, AIS has predefined message types and additional data can only be sent to another AIS transceiver using a couple of message types, in the form of binary data. In addition, a vessel first needs to reserve the AIS time slot(s) to send its message, which introduces additional management traffic and may take some time. We have not evaluated this communication bootstrapping approach.

#### 4.3.3. Decentralized Discovery

**Figure 6:**
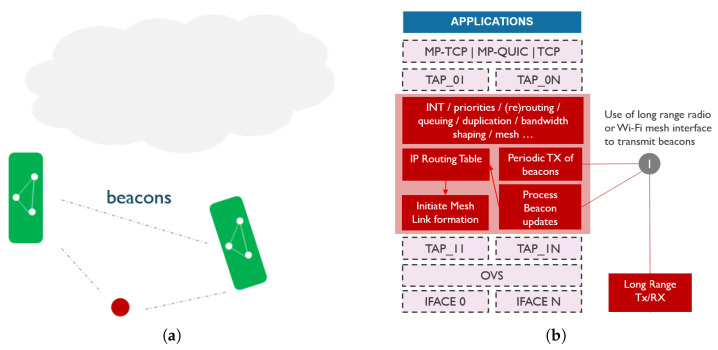
Decentralized discovery including (**a**) schematic representation of network topology and (**b**) communication stack design.

Decentralized discovery uses neither central server nor AIS. Instead, every vessel is assumed to be equipped with a Long-Range (LoRa) radio that broadcasts beacons periodically, as illustrated in Figure 6. Each beacon will contain bootstrapping information regarding the interfaces the vessel can support to connect to it. Given that long-range wireless technologies typically have a limited data rate too low to be used in critical scenarios, this technology may not be suitable for prolonged information exchange. However, vessels can exchange information on other communication capabilities via long-range communication technology, and establish a Wi-Fi mesh link for example to share more data.

### 4.4. Application-Network Integration

Application-network integration employed in this framework utilizes Application Network Agent (ANA) [4] and In-band Network Telemetry (INT) [19,25] for closing the loop between the applications and the underlying network. INT is used for real-time network performance monitoring on a per-hop and per-flow basis, whereas ANA is responsible for feeding back the INT metrics to the applications on one hand, and delivering the application requirements to the network on the other hand. Tighter application-network interaction leads to a better allocation of network resources for meeting application performance guarantees while making applications more adaptive to the underlying network context.

Application requirements are used both by ANA and by the abstraction layer. ANA instructs the monitoring stack which network metrics to collect (i.e., which parameters to monitor) based on the application requirements. For example, in presence of a latency requirement ANA will ask for monitoring of TX/RX timestamping at each network hop, whereas in presence of reliability requirement it will ask for monitoring packet loss on a per-hop basis. These INT measurements are passed to the application that could adjust its behavior based on the network conditions. The abstraction layer on the other hand uses application requirements to provide adequate quality of service. As such, the time-aware scheduler uses latency and Round Trip Time (RTT) requirements to schedule the queues. Reliability requirement, for example, could be met by triggering transmissions duplication in the abstraction layer over multiple communication links.

Application-network integration could enable local decision-making on whether and when to introduce new traffic flows (i.e., sensor streams). Based on the application requirements on one hand, and the INT data on the other hand, every node can be aware of the current state of the network and if there is enough bandwidth available to support a new traffic flow, considering its requirements.

## 5. Evaluation

This section details the evaluation of the three aspects of the proposed communication architecture:

- Communication stack performance,
- Communication bootstrapping, and
- Application-network integration.

### 5.1. Communication Stack Evaluation

The implemented abstraction layer depicted in Figure 1 operates in user space as opposed to the kernel stack. To avoid processing the traffic twice, in the kernel and user-level, we used OVS to bypass the part of the kernel stack and divert the traffic via the abstraction layer in user-level only. Regardless of the implementation in user-level, there are no additional delays or bottlenecks in the communication due to bypassing the kernel stack, and the implemented proof-of-concept that includes the abstraction layer performs nearly exactly the same as with the standard kernel stack, as shown in Table 1.

### 5.2. Communication Bootstrapping Evaluation

**Figure 7:**
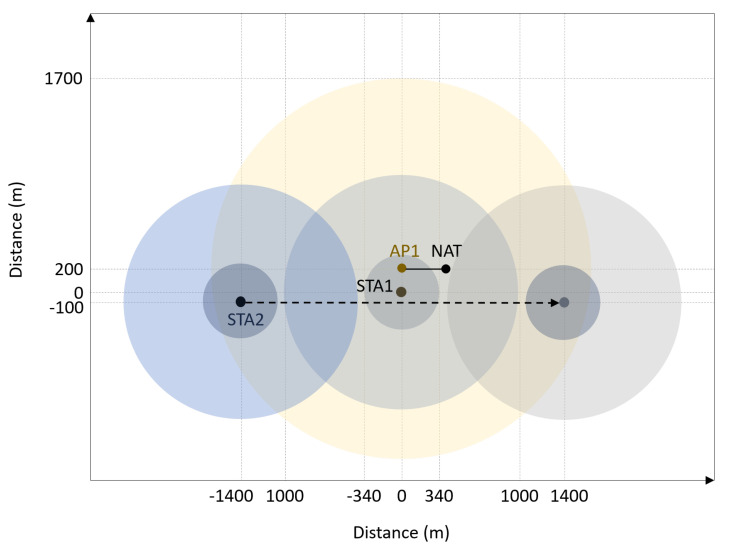
Visualization of the emulated network in communication bootstrapping evaluation, including mesh (small dark circle) and HaLow (larger dark circle) range of nodes.

Communication bootstrapping is evaluated in the scenario depicted in Figure 7 with two vessels, one static (STA1) and one mobile (STA2). Evaluation is done in Mininet-WiFi network emulator [26]. Given that Mininet-WiFi cannot emulate cellular networks, we used an Access Point (AP) with wide coverage (TX power of 35 dBm) in order to emulate a backbone cellular network. Network Address Translation (NAT) device is wired to the AP and serves as a gateway to the Internet where the central server is located. Hence, both vessels have Internet access over the Wi-Fi infrastructure network served by the AP, and make use of centralized discovery.

Each vessel is equipped with 4 Wi-Fi interfaces operating in 2.4 GHz band using 20 MHz-wide channels, out of which one IEEE 802.11g interface for utilizing the Wi-Fi infrastructure network served by the AP, two IEEE 802.11s mesh point Wi-Fi interfaces used for mesh communication bootstrapping (one for listening and one for transmitting) and one simulated Wi-Fi HaLow interface. Given that Mininet-WiFi does not support Wi-Fi HaLow (i.e., IEEE 802.11ah), by Wi-Fi HaLow in this paper we imply IEEE 802.11g in 2.4 GHz band with 30 dBm transmit power in order to simulate a 1 km link.

The mobile vessel moves at a constant speed of 55 km/h in a straight line past the static vessel, initially being in the range of the ap but out of both mesh and HaLow range of the static vessel. When the moving vessel arrives around 1 km far from the static vessel, centralized bootstrapping triggers a HaLow link establishment between the vessels. When the mobile vessel comes within 340 m of the static vessel, centralized bootstrapping also triggers mesh link establishment between the vessels. At this point vessels can communicate over three links, direct mesh link with most bandwidth, Wi-Fi Halow with mid-level bandwidth but longer range (up to 1 km), and backbone infrastructure network with the longest range but lowest bandwidth.

Both vessels publish their communication capabilities message (cf. Listing 1) whenever their configurations change. To reduce the network overhead, the location information is omitted from the communication capabilities message and published periodically every second in a shorter JSON structure containing only vessel’s MMSI and location information. Vessels consume the Kafka location messages every second and join the network of the remote vessel once the location of the remote vessel is within range of themselves. Table 2 summarizes the configuration of the experiment.

**Figure 8:**
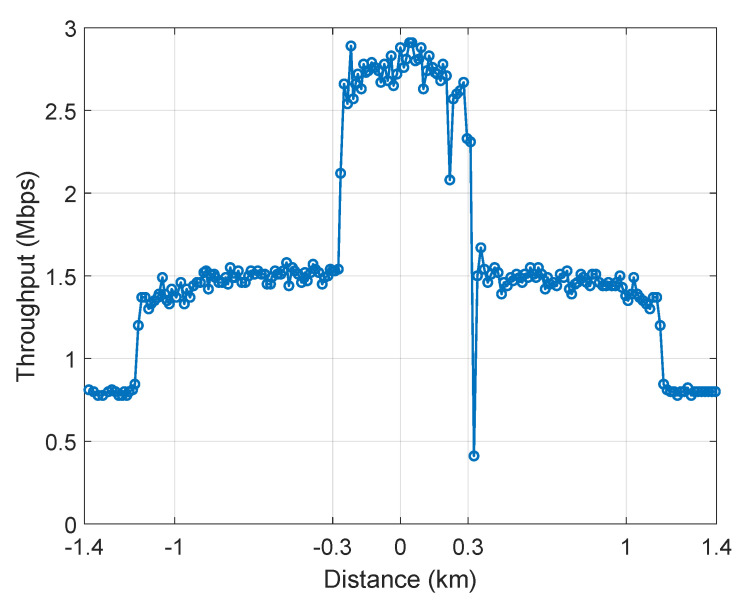
MPTCP throughput of mobile vessel STA2 using all available wireless links.

To demonstrate the benefit of multimodal communications, we used MultiPath Transmission Control Protocol (MPTCP) [27] to measure the network throughput in the scenario illustrated in Figure 7. Initially, both vessels use the infrastructure network to communicate with limited throughput (cca. 800 kbps). When STA2 arrives within the HaLow range of STA1, the abstraction layer triggers bootstrapping. Once the HaLow link has been established, MPTCP detects the new path and starts using it to get more throughput overall (cca. 1.5 Mbps), without breaking the TCP connection. This enables vessels in the vicinity of each other to exchange more data or use more services. Further, when STA2 arrives within the mesh range of STA1, the abstraction layer bootstraps the mesh communication as well, enabling the third link and providing even more throughput (cca. 2.8 Mbps). MPTCP iperf server was run on STA2, and client on STA1. Figure 8 shows how the overall throughput of the mobile vessel STA2 changes with the distance in the described scenario. The throughput and distance values are sampled once per second.

**Figure 9:**
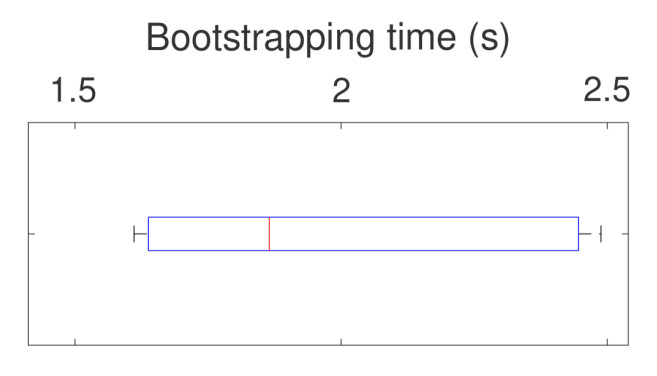
Distribution of time to bootstrap communication with centralized discovery using message update interval of 1 s.

To realize the JSON message exchange for centralized discovery, we used the Apache Kafka event streaming platform. A Kafka client was implemented in Click using the librdkafka C/C++ library, and we used Kafka cluster in the cloud (https://www.cloudkarafka.com/ accessed on 27 September 2021). Bootstrapping the mesh communication via Kafka cluster took 1.97 s on average over 10 runs, with an update interval of 1 s. Bootstrapping time distribution over 10 runs is shown in Figure 9. To establish a new link, nodes need to publish the most recent location information to the central server, consume the update from the peers and reconfigure their (TX mesh) interfaces (i.e., change the channel and reset the radio). Bootstrapping time is always less than 2.5 s which is sufficiently quick for mostly inert maritime vessels.

For the decentralized bootstrapping case, bootstrapping information is exchanged using periodic beacons sent by LoRa radios. As LoRa does not need any connection setup, it is a suitable technology for broadcasting bootstrapping information in a decentralized fashion. The only limitation will be the Maximum Transmission Unit (MTU) allowed and the data rate used. The MTU is subject to the data rate used and region. For EU863-870 MHz bands the mtu ranges from 59 B for DR0-DR2, 123 B for DR3 and 250 B for DR4 and DR5 [28].

The minimal settings that one vessel needs to broadcast are its receiver interface capabilities. As such, we have shrunk the JSON data structure from Listing 1 to include only the receiver interface capabilities and configuration, and Concise Binary Object Representation (CBOR) encoded it in order to fit to one 230 B LoRa MTU. We have calculated the transmission time for the different bands and regions and the results are shown in Table 3, where only the data rates that can support 230 B MTU are considered. We see that the largest beacon period is 64.5 s when lower data rates are used. Still such beacon periods will be acceptable considering the coverage areas that can be covered with lower data rates that can go up to several kilometers.

As evident from Table 3, message periodicity is limited by the beacon period. For the largest beacon period of 64.56 s, message TX time is 650 ms which means that the nodes can get the information needed for bootstrapping in time ranging from 0.65 s to 64.56 s. Although 64.56 s seems like a long time, note that this beacon period is used with DR4 that can cover the range of several kilometers, so that nodes quite far from each other would be able to discover each other and exchange the bootstrapping data (e.g., for Wi-Fi mesh) well before they arrive into each other’s Wi-Fi range.

### 5.3. Evaluation of Application-Network Integration

Application-network integration is evaluated in two scenarios, network-aware application scenario in Section 5.3.1, and application-aware network scenario in Section 5.3.2. In the former, network-aware application adjusts to the network conditions in real-time, based on end-to-end network measurements collected via INT. In the latter, the network adjusts to the applications’ requirements using ANA and the time-aware scheduling mechanism. Evaluation is done in an ad hoc Wi-Fi network (IEEE 802.11g) emulated in Mininet-WiFi [26]. Default wireless properties of the nodes are listed in Table 4.

#### 5.3.1. Network-Aware Application Scenario

To demonstrate the performance of a network-aware application, we emulated an IEEE 802.11g ad hoc network consisting of 3 nodes: A, B, and C. Node A represents a sensor on a vessel that samples every *x* ms and shares the measurement with node B on another vessel. The sampling rate, and thus the number of packets being generated by node A is adjusted to the network so that node B can receive all packets in a timely fashion, eventually process them and reply to node A. To model this, we used User Datagram Protocol (UDP) echo applications where node A periodically sends UDP packets to B, and B echoes every received packet to A. However, we limited the data rate of node B to half the value of A’s data rate. This means that if A samples too frequently and sends too much traffic out to B, B will not be able to receive, process, and reply to every packet in a timely fashion. Table 5 summarizes the parameters used in the emulation.

**Figure 10:**
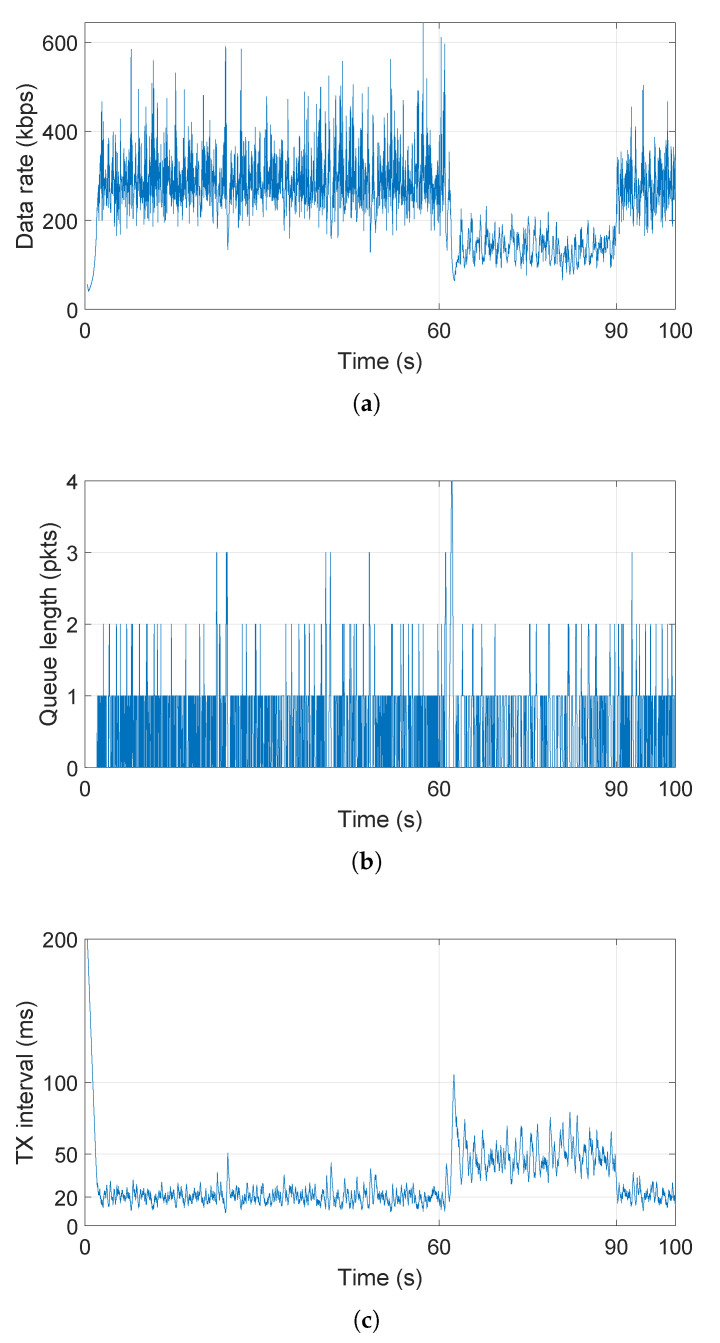
Network-aware UDP application’s (**a**) data rate, (**b**) queue length on the other end as indicated in received INT report and (**c**) transmission interval.

In order to adjust to the network (i.e., capabilities of the destination), node A’s application is INT-enabled, i.e., it receives and processes INT measurements from the network. From an INT report, node A’s application learns about the queue length status of the other end (node B) and can adjust its sampling rate in order to maximize the throughput, while making sure all transmitted packets are echoed by the endpoint. Initially, node A’s application starts sending data with TX interval of 200 ms and reduces it by 10% whenever an empty queue is detected on the other end in the received INT report, whereas it increases it for 10%, 20% and 40% when 1, 2 through 5, and 6 and more packets are indicated in the queue length field of the INT report, respectively. As shown in Figure 10, the TX interval of node A quickly converges to the value of 20 ms (i.e., max data rate of node B). At time 60 s, node B starts another UDP client application that sends 938 B every 30 ms to node C. This new application also fills node B’s outgoing queue, which is reflected in a peak in detected queue length in INT reports at node A (cf. Figure 10b), and in turn motivates node A to reduce its sampling rate, hence transmit less frequently. At time 90 s, the other application at node B is stopped, which is detected by node A via INT and node A again maximizes the throughput by increasing the sampling rate.

#### 5.3.2. Application-Aware Network Scenario

**Figure 11:**
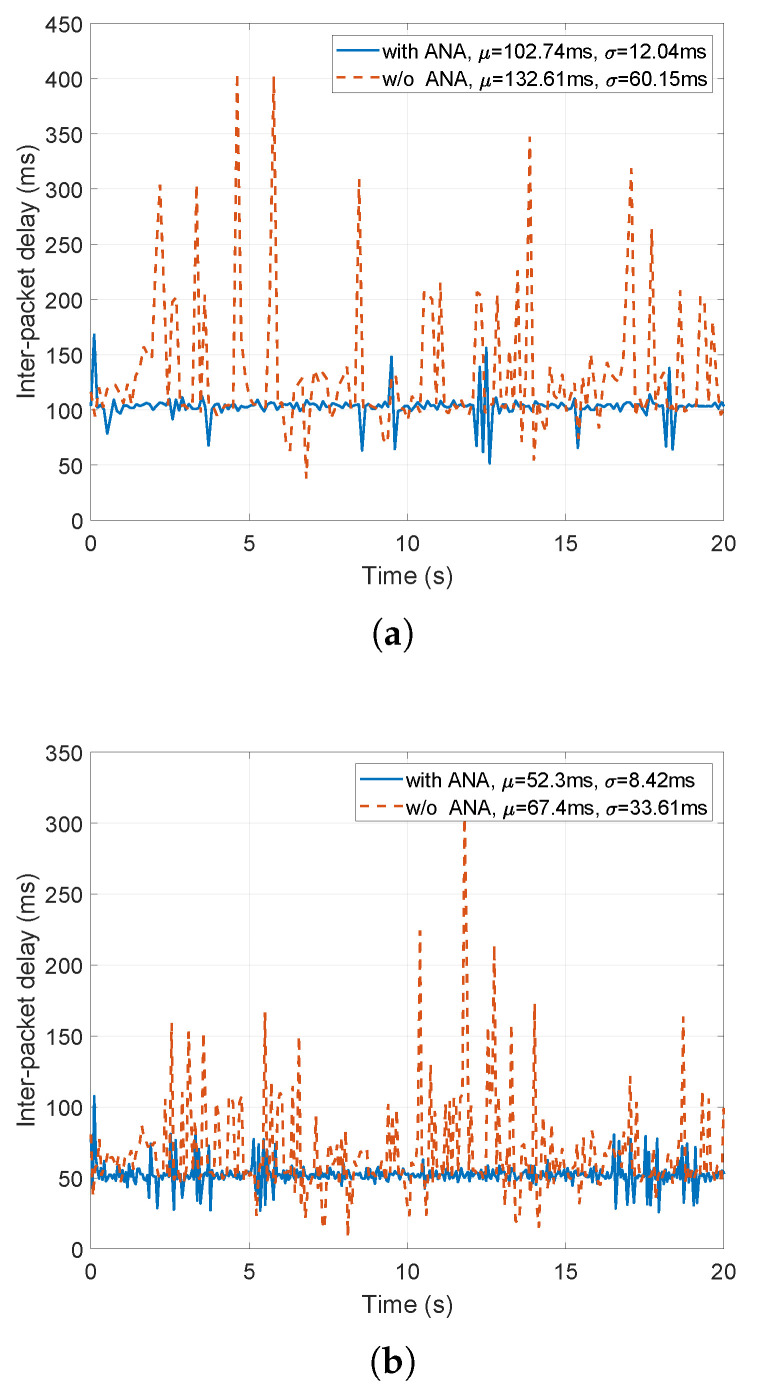
Performance of two simultaneously running UDP applications that require transmission delay less than (**a**) 100 ms (i.e., inter-packet delay < 200 ms) and (**b**) 50 ms (i.e., inter-packet delay < 100 ms) from a saturated IEEE 802.11g network.

In this scenario, two ad hoc IEEE 802.11g nodes placed 30 m far from each other communicate via UDP. One node runs simple UDP client applications, whereas the other runs UDP server applications. TX bitrates of both nodes are limited to 5.5 Mbps. Client node publishes application requirements to the ANA for 2 UDP applications, one transmitting 938 B of data every 50 ms, and the other transmitting 938 B every 100ms. In the application requirements, each client application specifies its data period and payload, as well as required application-layer latency equal to its data period. This means that every packet in the outgoing queue should be dequeued (i.e., transmitted) before the application enqueues the next packet. The abstraction layer employs the time-aware scheduler (cf. Section 4.2) in order to satisfy the application requirements. The client node also runs a best-effort logging application that attempts to use all available bandwidth. Figure 11 shows the inter-packet delays at the server side with and without the presence of the time-aware scheduler that handles the application requirements.

As evident from Figure 11, the application-requirements enabled time-aware scheduler manages to schedule all outgoing packets in the required time, hence causing a timely reception at the destination. Some additional delay may be present at the beginning of the emulation (for the first few packets) until the time-aware scheduler learns about the traffic patterns in the network and reconfigures itself according to the requirements and current traffic patterns. Moreover, in the scenario without ANA, all three applications (i.e., two applications with ANA and the best-effort logging application) experienced about 20% of packet loss. In the scenario with ANA where two applications require their needs to be met, they experience no packet loss, whereas the best-effort logging application experiences 30% of packet loss.

## 6. Conclusions

This paper presents the first multimodal and modular communication solution for enhancing situational awareness between vessels in the maritime domain. The solution proposed is not dependent on any particular communication technology, and can make use of all available communication interfaces present on a vessel. We designed, implemented and evaluated an abstraction layer located between layers 2 and 3 of the communication stack. The abstraction layer handles multimodal communications, including fully distributed communication bootstrapping, and improves the quality of service, transparently to the higher layer applications. This solution is modular enough to incorporate both existing and upcoming new communication technologies when they appear on the market. Finally, the proposed solution utilizes the state of the art INT [19] and ANA [4] frameworks to provide relevant network metrics to the applications as feedback, and to communicate application requirements to the network, without introducing additional management traffic and keeping the overhead limited.

The proposed solution is evaluated in terms of architecture performance as compared to Linux stack, communication bootstrapping, and application-network interaction scenario. The proposed architecture introduces virtually no additional delays as compared to the Linux stack. Automatic communication bootstrapping in a centralized scenario takes around 2 s, assuming the location updates to the central server of 1 s. In a decentralized scenario using LoRa radios to exchange the bootstrapping information, communication bootstrapping time is limited by the beacon period, but this limitation should not be crippling for the majority of use cases. Automatic communication bootstrapping enables the applications to make use of more bandwidth in a transparent fashion, without the need to explicitly reconnect and reroute the traffic over a better link once it becomes available. Finally, application-network integration enables the network to adjust to the application requirements as shown in the application-aware network scenario. On the other hand, INT enables the applications to adjust the applications’ behavior based on real-time network performance (e.g., data compression, filtering, enabling/disabling new streams, etc.), as shown in the network-aware application scenario.

## Figures and Tables

**Table 1:** Performance of the proposed communication architecture with OVS and the abstraction layer, versus Linux stack over ethernet measured with iperf (TCP).

Metric	Proposed Architecture	Linux Stack
Data transferred in 10 s [GB]	1.10	1.11
Number of packets	802,479	809,706
Throughput [Mbps]	944	952
Packet loss [%]	0	0

**Table 2:** Default parameters used in communication bootstrapping evaluation.

Parameter	Value		
Wi-Fi Mode	Managed	Mesh	HaLow
Propagation loss model	Log distance, exp = 3		
Coverage radius [m]	1578	340	1057
Max TX bitrate [Mbps]	1	2	1
Wi-Fi channel	1	6 and 7	11
TX power of vessel [dBm]	35	15	30

**Table 3:** Bootstrapping information transmission time and their minimal periodicity for different regions and channel bands.

Region/Bands	DR	MTU	Modulation	TX Time [s]	Beacon Period [s] *
EU863- 870 MHz					
EU 433 MHz	DR4	250	SF8	0.65	64.56
CN779- 787 MHz	DR5	250	SF7	0.36	36.37
CN470- 510 MHz	DR6	250	SF7	0.18	18.2
AS 923 MHz	DR7	250	FSK	0.05	5.12
KR920- 923 MHz					
IN865- 867 MHz					
RU864- 870 MHz					
US902-928	DR3 DR4 DR10 DR11 DR12 DR13	250 250 250 250 250 250	SF8 SF7 SF7 FSK	0.63 0.16 0.52 0.29 0.16 0.09	63.68 16.14 52.27 28.69 16.14 0.09
AU915-928	DR4 DR5 DR6 DR10 DR11 DR12 DR13	250 250 250 250 250 250 250	SF8 SF7 SF7 FSK	0.65 0.64 0.16 0.52 0.29 0.16 0.09	64.56 63.68 16.14 52.27 28.69 16.14 0.09

* The beacon period is calculated based on the duty cycle rule. For bands where no duty cycle rule; applies, transmission can happen more often applying listen before talk (LBT) rule only.

**Table 4:** Default parameters used in application-network integration evaluation.

Parameter	Value
Mode	ad hoc
Frequency	2412 MHz
Channel	1
Channel width	20 MHz
TX power	15 dBm
Bitrate	5.5 Mbps

**Table 5:** Default parameters used in network-aware UDP application evaluation.

Parameter		Value	
Node	A	B	C
INT-enabled	yes	no	no
INT period	100 ms	-	-
Max data rate [kbps]	750.4	375.2	-
Payload size [B]	938	938	938
TX interval	adjustable	echoed A	30 ms

## Data Availability

The data presented in this study are available on request from the corresponding author. The data are not publicly available due to intellectual property reasons.
